# Supplementary material for: The effects of Urban Identity on Entrepreneurial Choice
Source: PLoS One. 2024 Apr 3;19(4):e0296645. doi: 10.1371/journal.pone.0296645 (PMC10990197; doi:10.1371/journal.pone.0296645)
Supplement: Attachment — Submitted filename: Response to Reviewers.pdf [file pone.0296645.s002.pdf]

Response to Reviewers

1:The relationship between the identity and behaviour has long been a key question within social psychology and has recently received attention within the entrepreneurship domain . An identity provides an individual with a frame of reference with which to interpret both the social situation and his/her potential actions.As the highest level of social integration,the identity includes the integration of local society, economy and psychology. The UI can effectively enhance their cultural integration and reduce regional discrimination,and then reduce entrepreneurial barriers and transaction costs in the process of entrepreneurship.Further the UI promotes the reconstruction of the social networks and embeds it in the local social network, which is conducive to reducing information asymmetry, discovering entrepreneurial opportunities and increasing the possibility of financial lending; Starting a business from a psychological point of view gives the migrants an identity symbol to show their social status.It is in line with the ideal career orientation of urban people.

2: Probit model was put forward by American statistician C.R. Probit in 1958. The dependent variable of this model is binary, that is, there are only two possible outcomes: "Yes" or "No", but the independent variable can be continuous or classified. It assumes that there is a linear relationship between the probability of the dependent variable and independent variables, and then infers the degree of influence of different independent variables on the dependent variable.

3:The fair access of the migrants to public services, such as social management services and public health services, is binding in a certain extent. Similar circumstances inhibit the flow of identity of the migrants. The migrants admire the urban people included in community management,resulting in an independent sense of identity division.The convenience and availability of public services have a positive impact on the identity of the migrants .The establishment of health records for the migrants in communities can provide them with continuous, comprehensive, economic and scientific public health services and basic medical services, reflecting the public services integration of the migrants into cities.Obviously, the availability of public services affects the UI and residence intention of the migrants, is not related to their employment decisions.Therefore, we believe that the availability of public services is a reasonable instrumental variable.we select the proxy variable of the availability of public services based on questionnaire "whether the local community established a resident health record card for you".

4:Promote urban Cultural Integration

Articles identify the following most common challenges refugee and immigrants entrepreneurs encounter: (a) seed capital, (b) language barriers, (c) location, (d) embeddedness,(e) knowledge about the local market. The important connotation of identity is the individual's recognition of the identity norms to which they belong and Cultural Integration. It is manifested in the adaptation to the culture of the inflow area.The UI of the migrants promotes them to follow the social norms of the local people,and strengthen others' cognition of their own identity by imitating the behavior of the local people,such as similar in language use, customs, values, housing integration,

etc.The UI can reduce the conflict between the behavior norms of the migrants and the local people, reduce the discrimination in the inflow place, narrow the psychological distance .Further, the above behaviors lead to the preference of the inner group and increase the cooperative behavior of the members in the group.So the UI of the migrants can help alleviate the financial constraints and language barriers faced by the migrants in urban entrepreneurship, better embed in the local market and reduce friction and entrepreneurial barriers. As a whole, urban cultural Integration expand the expected income of entrepreneurship, and then enhance the entrepreneurial behaviors .

5:Dear editor, we have carefully studied your article on Initial Coin Offerings and Entrepreneurial Finance. There are 6 tables in the article, and the notes of each table elaborate the table variables and the meaning of the results, which makes the reading more clear. It makes the table more clear to the reader. However, the specific format of the table does not meet the requirements of plos one journal. I will use it in my next paper.
